# Living evidence and adaptive policy: perfect partners?

**DOI:** 10.1186/s12961-023-01085-4

**Published:** 2023-12-18

**Authors:** Tari Turner, John N. Lavis, Jeremy M. Grimshaw, Sally Green, Julian Elliott

**Affiliations:** 1 School of Public Health and Preventive Medicine, Monash University, Melbourne, Australia; 2 McMaster Health Forum, McMaster University, 1280 Main Street West, Hamilton, ON L8S 4L6 Canada; 3 Department of Health Research Methods, Evidence and Impact, McMaster University, 1280 Main St. West, Hamilton, ON L8S 4K1 Canada; 4 Centre for Health Economics and Policy Analysis, McMaster University, 1280 Main St. West, Hamilton, ON L8S 4K1 Canada; 5 Health Policy PhD Program, McMaster University, 1280 Main St. West, Hamilton, ON L8S 4K1 Canada; 6 Department of Political Science, McMaster University, Hamilton, Canada; 7 Africa Centre for Evidence, University of Johannesburg, Johannesburg, South Africa; 8 Ottawa Hospital Research Institute, Ottawa, ON Canada; 9 School of Population and Public Health, University of Ottawa, Ottawa, ON Canada; 10 Department of Medicine, University of Ottawa, Ottawa, ON Canada

**Keywords:** Evidence, Policy, Evidence-informed, Evidence-based, Living, Up-to-date, Research, Knowledge translation

## Abstract

**Background:**

While there has been widespread global acceptance of the importance of evidence-informed policy, many opportunities to inform health policy with research are missed, often because of a mismatch between when and where reliable evidence is needed, and when and where it is available. ‘Living evidence’ is an approach where systematic evidence syntheses (e.g. living reviews, living guidelines, living policy briefs, etc.) are continually updated to incorporate new relevant evidence as it becomes available. Living evidence approaches have the potential to overcome a major barrier to evidence-informed policy, making up-to-date systematic summaries of policy-relevant research available at any time that policy-makers need them. These approaches are likely to be particularly beneficial given increasing calls for policy that is responsive, and rapidly adaptive to changes in the policy context.

We describe the opportunities presented by living evidence for evidence-informed policy-making and highlight areas for further exploration.

**Discussion:**

There are several elements of living approaches to evidence synthesis that might support increased and improved use of evidence to inform policy. Reviews are explicitly prioritised to be ‘living’ by partnerships between policy-makers and researchers based on relevance to decision-making, as well as uncertainty of existing evidence, and likelihood that new evidence will arise. The ongoing nature of the work means evidence synthesis teams can be dynamic and engage with policy-makers in a variety of ways over time; and synthesis topics, questions and methods can be adapted as policy interests or contextual factors shift. Policy-makers can sign-up to be notified when relevant new evidence is found, and can be confident that living syntheses are up-to-date and contain all research whenever they access them. The always up-to-date nature of living evidence syntheses means producers can rapidly demonstrate availability of relevant, reliable evidence when it is needed, addressing a frequently cited barrier to evidence-informed policymaking.

**Conclusions:**

While there are challenges to be overcome, living evidence provides opportunities to enable policy-makers to access up-to-date evidence whenever they need it and also enable researchers to respond to the issues of the day with up-to-date research; and update policy-makers on changes in the evidence base as they arise. It also provides an opportunity to build flexible partnerships between researchers and policy-makers to ensure that evidence syntheses reflect the changing needs of policy-makers.

## Background

Evidence-informed policy is “an approach to policy decisions that aims to ensure that decision making is well-informed by the best available research evidence. It is also characterised by the systematic and transparent efforts to acquire, assess, adapt and apply evidence as part of the policymaking process.” [1].

While sometimes challenged, there has been widespread global acceptance of the importance of evidence-informed policy [2–5]. It is also widely recognised that many opportunities to inform health policy with evidence from research continue to be missed [6–8].

A number of barriers have been identified to the use of research evidence to inform health policy, among the most important being timely availability of reliable, relevant synthesised [9]. In response, there has been a growing emphasis on tailored knowledge translation activities to support the use of evidence in policy [10, 11].

A key principle of knowledge translation to support evidence-informed policy-making is that the focus should be on translating bodies of research, meaning syntheses of all the research addressing a given question, rather than the results of individual studies [11]. However conducting reliable syntheses, or systematic reviews, of research evidence can be time consuming, heightening barriers related to the timely availability of research evidence.

‘Living evidence’ focuses on ensuring that evidence products such as systematic reviews, clinical practice guidelines, and other evidence syntheses are continually updated to ensure they always reflect the entire body of evidence. Living evidence approaches are particularly valuable where: the evidence products address priority questions for decision-makers; the current evidence-base does not yet provide certainty; and it is expected that there will be a flow of new research which might affect the conclusions of the review [12]. Here we focus on ‘living systematic reviews’, in which systematic, rigorous evidence syntheses are continually updated to reflect new relevant evidence as it becomes available [13]. However, while the details of the processes vary with the type of evidence synthesis being made ‘living’ (see for example, the Australian living guidelines for the clinical care of people with COVID-19 [14], or the COVID-END [15]), most of the concepts we discuss apply to all living evidence synthesis products.

In each case, new technologies like machine learning, and new approaches to involving large groups of people, such crowd-sourcing, can substantially reduce the burden of time-consuming tasks like evidence screening [16–20], meaning it is now feasible to produce living systematic reviews. These living syntheses maintain the underlying rigorous systematic review methods that ensure they are reliable and harness new methods to ensure they are also always up-to-date.

The feasibility of applying living evidence approaches to producing living systematic reviews and living guidelines has been demonstrated by several pilot projects [21–24]. Living systematic reviews are now a core product in The Cochrane Library, and the methods that underpin them, such as machine learning and crowd-sourcing to improve efficiency of evidence identification have now been incorporated into standard Cochrane workflows [25, 26].

The value of living evidence syntheses in guiding clinical practice decisions as been emphasised by the need for rapid, reliable evidence to inform health decisions during the COVID-19 pandemic, and several teams, including the World Health Organisation, the Australian National COVID-19 Clinical Evidence Taskforce, COVID-END have applied these methods to provide living systematic reviews and guidelines to inform critical health decisions [14, 15, 27].

We believe that living evidence approaches are likely to have major benefits for policymaking too, a perspective that is shared by others including the Global Commission on Evidence to Address Societal Challenges [28]. Specifically, living evidence approaches have the potential to overcome a major barrier to evidence-informed policy, enabling reliable, up-to-date systematic summaries of policy-relevant research to be available at the time that policymakers need them. These approaches may be particularly important and useful for health policy now, given the increasing calls for adaptive, responsive, rapid learning approaches to health policy development and implementation.

The contemporary feasibility to produce living systematic reviews is matched by the receptivity of health policymakers to this very approach. Adaptive approaches to policymaking are increasingly being used in recognition of the uncertain, dynamic and complex nature of the policy environment. They include a commitment to and methods for revision and update of policy in response to changes over time; and seek to embed learning processes as part of policy implementation, the results of which are then used to inform iterative revisions to policy [29–32].

## Objective

In this paper we describe the opportunities presented by living evidence for evidence-informed adaptive policy-making; reflect on the challenges it faces and poses; and highlight areas for further exploration.

## Opportunities for living evidence to support policy-making

**Fig. 1:**
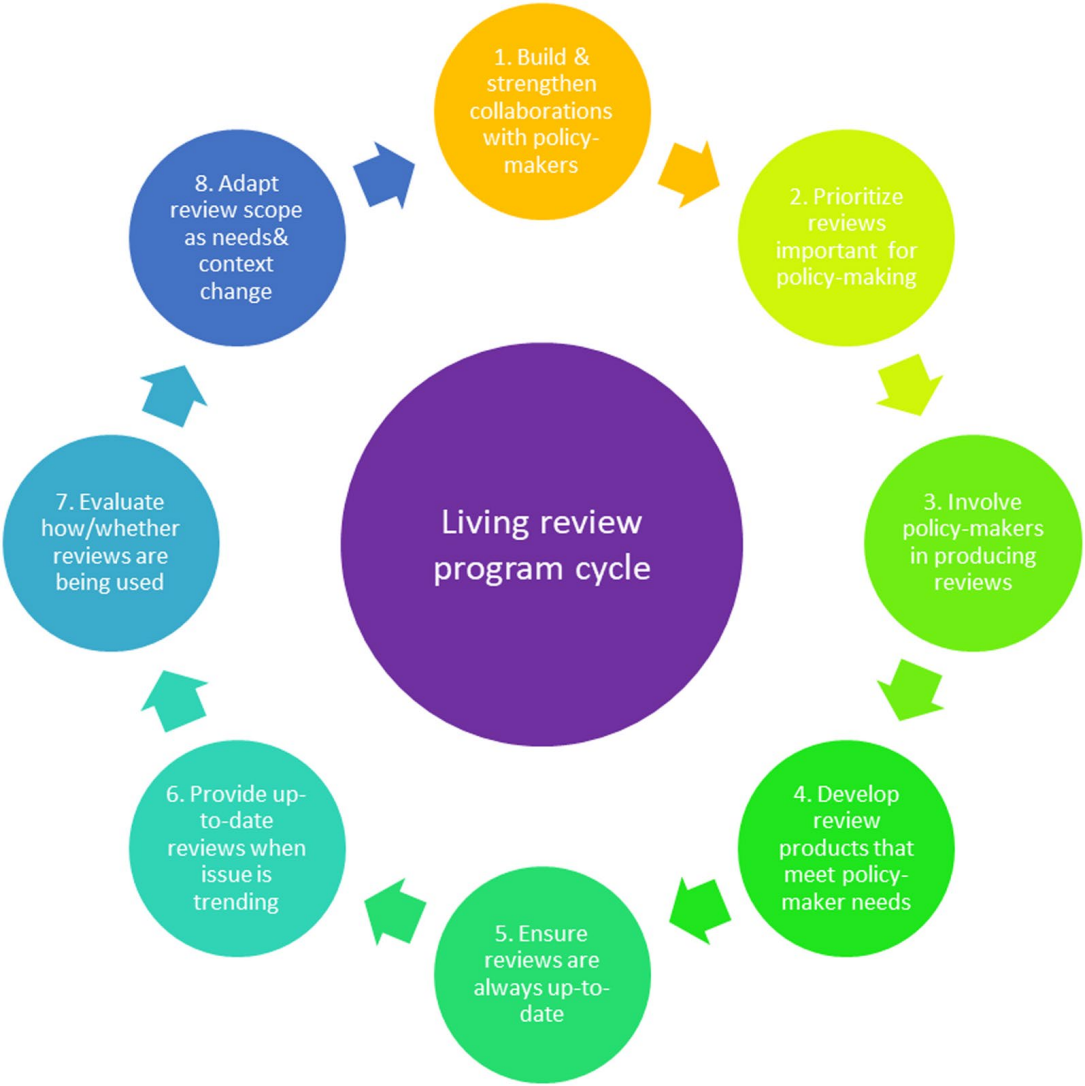
Living approaches applied to a systematic review program cycle

There are several ways in which ‘living evidence’ approaches (see Fig. 1) might overcome some of the key challenges to evidence-informed policy.

In Table 1 below, we outline some of the opportunities for living evidence to support evidence-informed policy-making. We have mapped these opportunities to the Knowledge Translation Framework developed by Cochrane [33] to guide knowledge translation activities that support use of evidence syntheses by health decision-makers. Designed by a group of knowledge translation experts both internal and external to Cochrane, it describes five areas of knowledge translation activity, and we believe there are opportunities for living evidence to support use of evidence to inform policy-making in each theme. It was also designed to apply to a number of audiences. We have tailored it slightly here to focus on policy-makers, one of Cochrane’s four target audiences.

**Table 1:** Potential opportunities presented by living evidence syntheses for adaptive policy

Theme from Cochrane KT Framework	Potential opportunities presented by living evidence
1. Prioritization and co-production of reviews—Producing reviews which meet the needs of policy-makers	• Policy makers can shape reviews to meet their needs as these vary over time • Syntheses which are high priority for policy-making are selected to be ‘living’ where they address priority questions for policymakers, there is uncertainty in the existing evidence, and it is expected that new evidence will arise and could change the conclusion of the review • Evidence teams can be dynamic and include, or engage with, the same or different policy-makers in a variety of ways over time, as questions and interests change
2. Packaging, push and support to implementation—Ensuring policy-makers receive and can act on reviews and products	• A ‘news feed’ of updated summaries of evidence in specific topics can be disseminated regularly through social and other media • Policy-makers can sign-up to be notified when important new evidence is identified in their topics of interest, or researchers can forward to policy-makers with known areas of interest
3. Facilitating pull—Growing policy-makers’ capacity to find and use reviews	• Policy-makers can be confident that reviews are both reliable and up-to-date, containing and appraising all relevant research, whenever they access them
4. Exchange—Engaging with policy-makers to support their evidence informed decision making	• Ongoing nature of production enables a stronger relationship between reviewers and policy-makers leading to better understanding of needs • Topics and questions can be revised and adapted as policy interests or contextual factors evolve over time
5. Improving climate—Advocating for evidence informed health policy-making	• Always up-to-date nature of reviews means review producers can rapidly disseminated and demonstrate availability of relevant, reliable evidence when issues gain policy interest, addressing a frequently cited barrier to evidence-informed policymaking and improving the climate for evidence use

### Prioritization and co-production—producing evidence syntheses which meet the needs of policy-makers

Living approaches make the key role of policy-makers in prioritising and producing living evidence syntheses explicit and flexible.

#### Reviews are selected for living approaches based on relevance to policy decision-making

Adoption of a living evidence approach for a particular evidence synthesis project is primarily based on an assessment of three criteria:

- that the question addressed by the review is of importance to decision-makers,
- that the existing evidence base doesn’t yet adequately answer the question,
- that more evidence is likely to become available to inform the question.

Making the importance of the question the first criterion ensures that the reviews prioritised for living approaches are those which will be useful to policy-makers.

#### Policy-makers contribute to producing reviews in flexible and dynamic ways

The ongoing, rather than one-off or project-based, nature of living approaches provides opportunities for partnerships between reviewers and policy-makers that are dynamic, flexible and enduring, enabling policy-makers to contribute to multiple cycles of the evidence review process, and to different stages of each cycle, from question setting and prioritisation, conduct and revisions and updates of evidence syntheses. The role of policy-makers might include true ‘co-production’ with policy-makers embedded in the living evidence synthesis team, provision of strategic guidance, contribution to question formulation and prioritisation, interpretation of findings, and can evolve over time as roles, interests and contexts change.

The rigorous, transparent, prespecified nature of the systematic reviews would be maintained, protecting against any concerns of selective evidence inclusion, or ‘policy-based evidence’[34].

Approaches to producing living syntheses might also be tailored to different types of policy; enabling differentiation between key policy areas which are likely to remain high priority, and in which evidence syntheses will always need to be maintained in living mode, and emerging policy interests where maintaining syntheses in a living mode may be appropriate for a particular time period. Recognising that some topics remain on the policy agenda for years (eg human health resources) or come up cyclically.

### Packaging, push and support to implementation—ensuring policy-makers receive and can act on reviews and products

Living approaches can effectively provide a news service, informing policy-makers when conclusions change.

#### Reviewers can provide rapid updates to interested policy-makers

Living evidence approaches result in opportunities to provide policy-makers who have interests in specific policy areas with updated evidence summaries as new evidence becomes available, allowing them to maintain a watching brief on areas of interest. This is particularly important when new evidence leads to changes in the interpretation or conclusions of the synthesis, with potential implications for policy decisions.

Policy-makers might choose to ‘subscribe’ to these updates, or researchers might proactively send them to policy-makers with known areas of interest. Policy-makers could then be confident that they are fully informed of the latest research.

### Facilitating pull—growing policy-makers’ capacity to find and use evidence

Lack of timely availability of syntheses of all relevant research evidence is a key barrier to evidence use in policy. When policy-makers need evidence, they typically need it within a time-limited window. However, producing or updating a traditional systematic review can at best take weeks, and often takes months or even years. As a result, ensuring evidence syntheses are up-to-date at the—often unpredictable—moment that policy-makers need them has been a major challenge. This is overcome by living approaches which can respond to ‘just-in-time pull’ by policy-makers.

#### Policy-makers can be confident that living reviews are up-to-date whenever they need them

Living evidence syntheses, which are continually maintained in an up-to-date state, with any new relevant research rapidly incorporated as soon as it becomes available, can provide reliable research evidence syntheses which are up-to-date whenever a policy-maker needs them.

Whenever a policy-maker accesses a living synthesis, they can be confident it includes all of the available, relevant, reliable evidence; and that they are fully informed.

Collecting and curating living syntheses in a dedicated database or in one of the existing databases, would also enable policy-makers to explore policy topic areas of interest in which new research evidence is emerging, and keep up-to-date with the changing evidence base over time, in a ‘grazing’ mode. Such a database could also reduce duplication in production of these syntheses.

### Exchange—engaging with policy-makers to support their evidence informed decision making

The ongoing nature of living synthesis production enables a continuing, flexible, enduring relationship between reviewers and policy-makers to better understand each other’s roles and needs.

#### Living reviews can respond to changing circumstances or needs

A living approach to production enables the product to be refined over time to reflect changes in areas of policy interest, or in the external environment. This adaptive approach is useful in areas where, for example, there are changes in the health system that affect policy implementation; or the funded patient population is being broadened for a particular intervention. Our experiences in preparing living evidence syntheses that were relevant to policy in the evolving context of new variants of SARS-COV-2 highlighted the value of this flexibility.

Living approaches are also likely to support conversations between policy-makers and researchers over time, to review the direction and relevance of evidence syntheses and to ensure that they continue to meet the policy-makers’ needs. Living reviews are also an optimal input into derivative products, such as living citizen briefs and living evidence briefs, that are used to inform citizen panels and stakeholder dialogues, which can support evidence-informed policymaking in specific contexts. Living reviews would always contain all the relevant evidence that these derivative products draw on, and could therefore be used at any time that suited the particular policy context.

### Improving climate—advocating for evidence informed health policy-making

Living approaches provide an opportunity to demonstrate the usefulness and responsiveness of research evidence to policy issues and increase trust in evidence informed policy making.

#### Synthesis producers can rapidly demonstrate availability of relevant, reliable evidence when issues gain policy interest

Living evidence models provide an opportunity to engage with policymakers in a window of opportunity for policy when an issue is ‘trending’, i.e. receiving increased global or local attention, via social and traditional media.

When maintaining a living evidence synthesis, researchers are ready to contribute research to inform policy discussions in a timely way because the evidence syntheses are always up-to-date. Researchers can, therefore, be responsive to rapidly increasing interest in an issue when it arises, broadcasting living evidence summaries to a broad policy audience through social and traditional media, news feeds, and via other communication channels, and making timely, reliable, up-to-date contributions to policy conversations initiated by others (such as those convening citizen panels and stakeholder dialogues). These approaches will require sophisticated, tailored horizon scanning in both research and policy fields.

Living approaches may also enable a more sophisticated communication about, and ideally growth in acceptance of, uncertainty; by explicitly articulating what we know and don’t know on the basis of available research at any point, and what has changed in the evidence about a policy question over a particular period of time.

## The future

Living approaches to evidence synthesis are in early phases of development, and most of the work so far has been in living systematic reviews about programs, services and products; and reviews for living clinical practice guidelines [12, 35], with a focus on clinical decision-making. This work suggests that it is likely that living approaches will benefit evidence-informed health policy-making. However, the value of living evidence is yet to be substantively tested in policy-relevant domains such as governance, financial and delivery arrangements that determine whether the right programs, services and products get to those who need them.

Living evidence models provide potential opportunities to improve prioritisation and co-production of reviews; to ensure policy-makers are informed of changes to reviews in their areas of interest as they occur; to build confidence that reviews will be up-to-date whenever they are needed; to allow flexible models of engagement between researchers and policy-makers; and to ensure reviews respond to changes in the external environment. These applications of living evidence encapsulate many of the ‘dos and don’ts of influencing policy’ identified by Oliver and Cairney in their 2019 systematic review [36], including making research relevant and readable; understanding policy processes; being accessible to policymakers: engaging routinely, flexible, and humbly; and building relationships (and ground rules) with policymakers. They also address several of the key barriers to policy use of evidence, primarily around timely supply of relevant, up-to-date, reliable evidence syntheses [9].

As we move into testing these living approaches to informing policy with synthesised evidence, several potential challenges are clear, and it is likely that others will emerge.

**Fig. 2:**
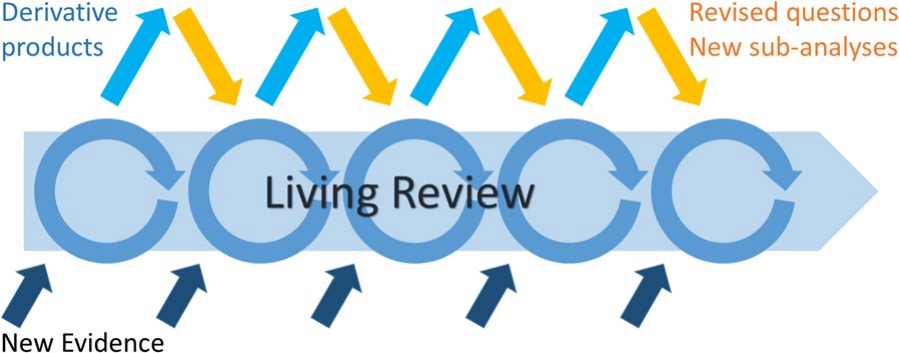
A living review process

The potential to revise, refine and refocus questions to reflect changes in policy context and areas of policy interest is a clear potential benefit of living syntheses over static, one-off methods, but how this will work in practice is not yet clear. Ensuring living systematic reviews evolve to continue to be relevant to policy will require evidence teams to have systems that enable them to be attentive to relevant shifts in the policy world, which is a complicated undertaking. Success will rely on direct, effective connections between reviewers and policymakers, and may only be possible in a limited or local context. One potential approach to handling this might be to develop a central living systematic review as a global public good, from which can be produced derivative products for particular policy contexts at specific points in time (see Fig. 2). The curators of the central living review would then need to make ongoing decisions about whether and how to incorporate the sub-analyses, scope changes or other revisions made in the derivative products.

The ability to incorporate changes in the evidence team over time has important potential benefits, but it also raises challenges to current models for making decisions about assigning authorship; academic incentives that reward citations for peer-reviewed journal publications; and publication models that are still based on static, individual outputs. It is likely that models for living evidence syntheses will continue to evolve in parallel with changes in these associated systems.

Similar, although perhaps more rapid, evolution is also underway in the technologies that enable living evidence syntheses. Existing machine learning tools have already demonstrated marked improvements in efficiency in key steps of the systematic review process [37, 38], and substantial further improvements are likely.

It is important to acknowledge that the living evidence synthesis model relies on the availability of resourcing, skills and capacity for the duration of the period that the synthesis remains in living mode. It is as yet unclear how many policy questions meet living evidence criteria and would benefit from living evidence approaches, for what periods of time and what the requisite workload would be. Funding models that address this will be key to realising the potential benefits of this approach.

## Conclusions

Lack of timely availability of relevant, reliable research syntheses is a major barrier to evidence-informed policy. Living evidence may provide a powerful tool to overcome this barrier, ensuring high-quality syntheses of policy-relevant research are continually updated, and always reflect the most recent research findings. By doing so, living evidence provides opportunities for policy-makers to access up-to-date evidence whenever they need it; enables researchers to respond to the issues of the day with up-to-date research; and updates policy-makers on changes in the evidence base as they arise. It also provides an opportunity to build flexible partnerships between researchers and policy-makers that can ensure that evidence syntheses reflect the changing needs of policy-makers and the changing world in which they are operating.

## Data Availability

Not applicable.
